# Risk factors for pegylated liposomal doxorubicin-induced moderate to severe hand-foot syndrome in breast cancer patients: assessment of baseline clinical parameters

**DOI:** 10.1186/s12885-021-08028-8

**Published:** 2021-04-07

**Authors:** Guohua Liang, Wenjie Ma, Yanfang Zhao, Eryu Liu, Xiaoyu Shan, Weiwei Ma, Dabei Tang, Liru Li, Xingjian Niu, Wenhui Zhao, Qingyuan Zhang

**Affiliations:** 1 Department of Medical Oncology, Harbin Medical University Cancer Hospital, Harbin, 150081 China; 2 Department of Medical Oncology, General Hospital of Heilongjiang Provincial Agricultural Reclamation Bureau, Harbin, 150081 China

**Keywords:** Breast Cancer, Pegylated liposomal doxorubicin, Hand-foot syndrome, Risk factors, Predictive model

## Abstract

**Background:**

Hand-foot syndrome (HFS) is a side effect of skin related to pegylated liposomal doxorubicin (PLD) application. Moderate to severe hand-foot syndrome (MSHFS) might have a serious impact on patients’ quality of life and treatment. However, information on risk factors for the development of MSHFS is still limited. To analyze the risk factors for PLD-induced MSHFS in breast cancer patients and constructed a logistic regression prediction model.

**Methods:**

We conducted a retrospective analysis of breast cancer patients who were treated with a PLD regimen in the Tumor Hospital of Harbin Medical University from January 2017 to August 2019. A total of 26 factors were collected from electronic medical records. Patients were divided into MSHFS (HFS > grade 1) and NMHFS (HFS ≤ grade 1) groups according to the NCI classification. Statistical analysis of these factors and the construction of a logistic regression prediction model based on risk factors.

**Results:**

A total of 44.7% (206/461) of patients developed MSHFS. The BMI, dose intensity, and baseline Alanine aminotransferase (ALT) and Aspartate aminotransferase (AST) levels in the MSHFS group, as well as good peripheral blood circulation, excessive sweat excretion, history of gallstones, and tumour- and HER2-positive percentages, were all higher than those in the NMHFS group (*P* < 0.05). The model for predicting the occurrence of MSHFS was *P* = 1/1 + exp. (11.138–0.110*BMI-0.234*dose intensity-0.018*baseline ALT+ 0.025*baseline AST-1.225*gallstone history-0.681* peripheral blood circulation-1.073*sweat excretion-0.364*with or without tumor-0.680*HER-2). The accuracy of the model was 72.5%, AUC = 0.791, and Hosmer-Lemeshow fit test *P* = 0.114 > 0.05.

**Conclusions:**

Nearly half of the patients developed MSHFS. The constructed prediction model may be valuable for predicting the occurrence of MSHFS in patients.

## Background

Breast cancer is the most prevalent malignancy in women worldwide [1]. It was estimated in 2018 that approximately 2.1 million new cases of women were diagnosed with breast cancer, accounting for almost 25% of female cancer cases [2]. Currently, systemic chemotherapy is still a common method of treating breast cancer. Anthracyclines, such as doxorubicin, represent one of the dominant therapeutic drugs [3]. The main limitation, however, is that the use of anthracyclines results in some life-threatening side effects; for example, cardiotoxicity is the most serious side effect of doxorubicin [4]. To reduce the side effects of anthracyclines and improve their efficacy, several studies have been carried out to find new strategies to maximize clinical efficacy and to simultaneously control the side effects of doxorubicin [5]. Therefore, PLD came into being; liposome preparation encapsulates doxorubicin in a microcapsule structure so that cardiomyocytes with weak phagocytosis cannot be absorbed, thus reducing the toxic side effects on the myocardium [6]. In addition, PLD has a good passive targeting effect, and its mechanism is to enhance the permeation and retention (EPR) effect, so the drug concentration of PLD in tumour tissue is dozens of times higher than that in normal tissue, thereby enhancing the anti-tumour effect of PLD. However, with the increasingly wide application of PLD in the clinic, the number of side effects of PLD-related chemotherapy for HFS has also increased.

Hand-foot syndrome (HFS), also known as palmar-plantar erythema, palmar-plantar erythrodysesthesia, or Burgdorf’s syndrome, is a relatively common skin reaction associated with multiple chemotherapeutic agents. Among them, the incidence of PLD-induced HFS is approximately 50% [7]. Clinically, mild HFS is characterized by mild skin changes or dermatitis, including erythema, oedema or hyperkeratosis, and generally is not associated with pain, while moderate and severe HFS is characterized by skin desquamation, chapped and indurated blisters or severe pain. Even though usually non-life-threatening, HFS might be the leading reason for reduced chemotherapy compliance, and moderate and severe hand-foot syndrome (MSHFS) might have a serious impact on patients’ quality of life, psychology, and treatment and may even impair survival benefits for patients.

Thus far, it is unclear which risk factors may be associated with MSHFS caused by the use of PLD in breast cancer. Therefore, we studied the risk factors for developing MSHFS in breast cancer patients who received PLD treatment and constructed a logistic regression prediction model to provide a theoretical and clinical basis for the prevention of MSHFS.

## Methods

### Patients

We performed a retrospective analysis of breast cancer patients who were treated with PLD neoadjuvant chemotherapy or adjuvant chemotherapy in the Tumour Hospital of Harbin Medical University from January 2017 to August 2019, and 461 eligible patients were included in the analysis. The inclusion criteria were as follows: 1) patients were confirmed to have breast cancer by pathology; 2) patients received DC or DC/T (H) adjuvant or neoadjuvant chemotherapy (D: pegylated liposomal doxorubicin injection dose intensity of 20–40 mg/m2); and 3) patients were female. The exclusion criteria were as follows: 1) simultaneous metastatic foci of other organs were found at the first diagnosis; 2) other malignant tumours had been diagnosed in the past 5 years, except for cured non-melanoma skin cancer and cervical carcinoma in situ; 3) patients who had been treated for recurrent breast cancer in the past; 4) patients with incomplete data; and 5) patients with a history of skin diseases, Peripheral venous thrombosis, diabetics with comorbidities and may affect peripheral blood circulation.

### Study methods

#### Data collection

We collected general information (age, BMI, ECOG score, peripheral blood circulation, the presence or absence of tumours, sweat excretion), previous disease history (hypertension, diabetes, cholecystitis, history of gallstones, viral hepatitis), data regarding chemotherapy (allergy during the first PLD infusion, PLD dose intensity), biological parameters (baseline ALT, baseline AST, baseline GGT, baseline TBIL, baseline monocyte absolute value (MONO), neutrophil-lymphocyte ratio (NLR), platelet-lymphocyte ratio (PLR), baseline CEA, baseline CA153), and pathological information (ER, PR, HER-2, KI67). In this study, peripheral blood circulation and sweat excretion were collected from clinical nursing record sheet.

#### Criteria for determining some research factors

1. HFS diagnostic criteria: HFS grading according to the NCICTCAEv4.0 grading criteria: grade 1: minimal skin changes or dermatitis (e.g., erythema, oedema, or hyperkeratosis) without pain; grade 2: skin changes (e.g., peeling, blisters, bleeding, oedema, or hyperkeratosis) with pain, limiting instrumental activities of daily life; and grade 3: severe skin changes (e.g., peeling, blisters, bleeding, oedema, or hyperkeratosis) with pain, limiting self-care activities of daily life [8].
2. Hormone receptor and other criteria: The status of ER, PR, HER2 and Ki-67 was determined according to the results of immunohistochemical detection in the pathology department of our hospital. ER ≥ 1% was defined as ER positive; PR ≥ 1% was defined as PR positive; HER2 positive was defined as HER2 (3 +) or HER2 (2 +) but fluorescence in situ hybridization (FISH) indicated positive patients; Ki67 > 20% was considered high expression, while Ki67 ≤ 20% was defined as low expression. This study is a retrospective clinical study. Because the patient did not undergo vascular ultrasound examination at baseline, this study used skin temperature check (Tskin-diff) to evaluate the quality of peripheral blood circulation. As follow, in the same environment for 10 min, the temperature gradient from the center area to the toe, and upper arm to fingertips is greater than 1 °C is defined as the peripheral blood circulation is poor, and 1 °C or less is the peripheral blood circulation is good. Excessive sweating in this study was determined based on the hyperhidrosis NCI CTCAE v4.0 grading criteria, Grade 1 and above is defined as excessive sweat. Grade 1 Limited to one area (palms, soles, armpits); personal hygiene care is required. Grade 2 More than one site; patients need medication; accompanied by psychological effects. Grade 3 Involves multiple areas, not limited to palms, soles, and underarms; accompanied by electrolyte/hemodynamic imbalance.

#### Grouping

According to the HFS NCI-CTCAE v4.0 grading standard, the patients with HFS ≤ 1 who did not need to adjust the PLD dose and the patients with unrestricted instrumental activity were defined as the NMHFS group (*n* = 255 cases); patients with HFS ≥ 2 who needed to adjust the PLD dose and who had the limitation of instrumental activity were divided into the MSHFS group (*n* = 206 cases).

### Statistical analysis

This study used IBM SPSS 22.0 statistical software for statistical analysis. First, the measurement data were tested for normality. The measurement data conformed to a normal distribution and are described by the mean ± standard deviation. The data were analysed by independent sample t-tests. The data with a skewness distribution or an unknown distribution are represented by the median and quartile spacing (M, IQR), and the data were analysed by the rank sum test. The classified data are expressed as n (%), and the differences between the two samples were analysed by the chi-square test. The risk factors selected by the single factor analysis were analysed by binary multivariate logistic regression, and the odds ratio (OR) and its 95% confidence interval (CI) were calculated. According to the sample multivariate logistic regression analysis, the regression prediction model was established, and the Hosmer-Lemeshow fit test was carried out. The area under the curve (AUC) of the receiver operating characteristic (ROC) curve for the prediction rate of the model was drawn to evaluate the validity of the prediction model.

## Results

### Patient information

**Fig. 1:**
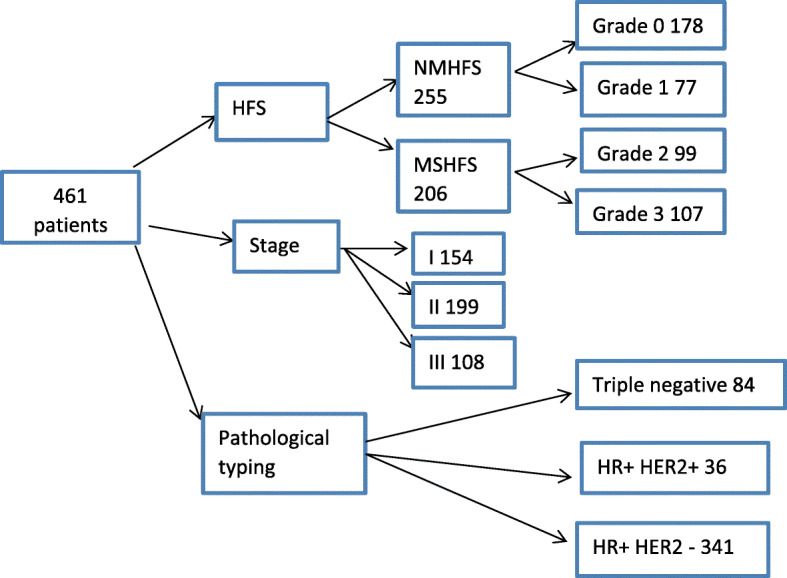
Basic patient characteristics

A total of 461 patients were included in this study. According to the NCI-CTCAE v4.0 HFS classification, there were 178 cases of HFS grade 0, 77 cases of grade 1, 99 cases of grade 2 and 107 cases of grade 3. There were 255 patients in the NMHFS group and 206 patients in the MSHFS group. The incidence of MSHFS was 44.7%. According to the AJCC staging standard: 154 persons in stage I; 199 persons in stage II; 108 persons in stage III. There were 84 patients with triple negative pathological classification; 36 patients with HR+ and HER2+; 341 patients with HR+ and HER2-.The mean age was 51.130 ± 9.565 years old. The median BMI score was 23.83 kg. There were 5 patients with an ECOG score of 0, 32 patients with diabetes, 45 patients with hypertension and 21 patients with a history of gallstones. There were 25 patients with a history of cholecystitis and 31 patients with a history of viral hepatitis. There were 339 patients with good peripheral blood circulation and 122 patients with poor peripheral blood circulation. There were 25 patients with a history of cholecystitis and 31 patients with a history of viral hepatitis. There were 339 patients with good peripheral blood circulation and 122 patients with poor peripheral blood circulation. ER was positive in 307 cases and negative in 157 cases, PR was positive in 266 cases and negative in 195 cases, HER2 was positive in 112 cases and negative in 349 cases, and there was high expression of Ki67 in 301 cases and low expression in 160 cases. The median dose intensity was 31.2 mg/m2, the median ALT was 15 U/L, the median AST was 19 U/L, the median GGT was 19 U/L, the median TBIL was 13 μmol/L, the median MONO was 0.37 × 109/L, the median NLR was 1.75, the median PLR was 132, the median CEA was 1.64 ng/ml, and the median CA153 was 9.2 ng/ml (Table 1, Fig. 1). In this study, during the course of treatment, patients had different adverse reactions. Among them, MSHFS was the side effect in 206 patients, neuropathy 101 patients, hepatotoxicity 98 patients, nephrotoxicity 2 patients, and alopecia 306 patients. All patients showed no cardiotoxicity. 12 patients discontinued PLD chemotherapy due to MSHFS and switched to adriamycin combined with cyclophosphamide chemotherapy. After changing the regimen, MSHFS was significantly relieved. Due to MSHFS, 89 patients delayed treatment for 5–7 days and 61 patients adjusted the dose to 75 to 80% of the initial dose (Table 2). Except for MSHFS, the other adverse reactions were all grade I-II, which did not meet the criteria for stopping or delaying treatment.

**Table 1:** Baseline and clinical characteristics collected from medical records

Factor	n(%)/x ®±S / M, Q25-Q75
Age	51.130 ± 9.565
BMI	23.83, 22.03–26.17
PLD dose intensity	31.20, 28.99–34.22
ECOG 1 Score	5 (1.1)
Hypertension disease	45 (9.8)
Diabetes	32 (6.9)
Cholecystitis	25 (5.4)
History of gallstones	21 (4.6)
Good peripheral blood circulation	339 (73.5)
Excessive sweat excretion	83 (18.0)
Allergic to the first infusion	23 (5.0)
Viral hepatitis	31 (6.7)
neoadjuvant treatment	128 (27.8)
ER+	307 (66.6)
PR+	266 (57.7)
HER-2+	112 (24.3)
KI-67 high expression	301 (65.3)
Baseline ALT	15, 12–21
Baseline AST	19, 17–23
Baseline GGT	19, 14–26
Baseline TBIL	13, 10–16.35
Baseline MONO	0.37, 0.305–0.45
Baseline NLR	1.75, 1.345–2.365
Baseline PLR	132, 108–170.5
Baseline CA153	9.2, 7–13.7
Baseline CEA	1.64, 1–2.5

KI-67 high expression: Ki67 > 20% was considered high expression

*Abbreviations*: *ALT* alanine aminotransferase, *AST* aspartate aminotransferase, *BMI* body mass index, *ECOG* eastern cooperative oncology group, *ER* estrogen receptor, *GGT* γ-glutamyl transpeptidase, *IQR* interquartile range, *HER2* human epidermalgrowth factor receptor-2, *Ki-67* antigen identified by monoclonal antibody Ki-67, *MSHFS* moderate to severe hand-foot syndrome, *MONO* monocytes, *NLR* neutrophil-lymphocyte ratio, *NMHFS* none and mild hand-foot syndrome, *PLR* platelet-lymphocyte ratio, *PR* progesterone receptor, *TBIL* total bilirubin

**Table 2:** PLD dose adjustment in MSHFS patients

MSHFS 206		Number
Therapy	Neoadjuvant therapy	67
	Adjuvant therapy	139
First cycle dose (mg)	70	3
	60–69	64
	50–59	113
	40–49	25
	< 39	1
MSHFS appearance cycle	1	18
	2	86
	3	78
	4	24
Cumulative dose at the time of MSHFS (mg)	> 199	20
	180–199	24
	160–179	18
	140–159	36
	120–139	26
	100–119	53
	80–99	11
	60–79	10
	40–59	8
Reduce the dose (%)	20%	42
	25%	19
Extension of time (Day)	7	33
	6	17
	5	39

*Abbreviations*: *MSHFS* moderate to severe hand-foot syndrome

### Results of single factor analysis

The median BMI in the MSHFS group was 24.154 kg/m2 (IQR 4.63), the median BMI in the NMHFS group was 23.440 kg/m2 (IQR 4.09), and *P* = 0.028; the median PLD dose intensity in the MSHFS group was 32.47 mg/m2 (IQR 5.205), the median PLD dose intensity in the NMHFS group was 30.18 mg/m2 (IQR 5.41), and *P* < 0.001; the median baseline ALT was 16 U/L in the MSHFS group (IQR 11), the median baseline ALT was 15 U/L (IQR 8) in the NMHFS group, and *P* = 0.012; and the median baseline AST was 20 U/L (IQR 7) in the MSHFS group, the median baseline AST in the NMHFS group was 19 U/L (IQR 5), and *P* = 0.023. Compared with the NMHFS group, the MSHFS group had a higher percentage of peripheral blood circulation [167 cases (65.5%) vs 171 cases (83.5%), P < 0.001], a higher percentage of excessive sweat excretion [28 cases (11.0%) vs 55 cases (26.7%), P < 0.001], a higher proportion of preoperative tumours [61 cases (23.9%) vs 67 cases (32.5%), *P* = 0.040], a higher HER2-positive rate [47 cases (18.4%) vs 65 cases (31.6%), *P* = 0.001], and was more likely to have a history of gallstones [5 cases (2.0%) vs 16 cases (7.8%), *P* = 0.003]. Age, ECOG score, hypertension, diabetes, cholecystitis, viral hepatitis, allergy to the first infusion of PLD, ER, PR, Ki67, baseline GGT, baseline TBIL, baseline MONO, baseline NLR, baseline PLR, baseline CEA, and baseline CA153 were not significantly different between the groups (*P* > 0.05, Table 3).

**Table 3:** Univariate analysis of MSHFS in patients undergoing PLD chemotherapy

Factor		NMHFS(n = 255)	MSHFS(n = 206)	*P*
Age		51.10 ± 9.94	51.17 ± 9.10	0.936
BMI		23.44, 21.61–25.71	24.154, 22.21–26.84	0.028*
PLD dose intensity		30.18, 26.79–32.20	32.47, 30.50–35.71	<0.001**
Baseline ALT		15, 11–19	16, 12–23	0.012*
Baseline AST		19, 17–22	20, 12–24	0.023*
Baseline GGT		18, 14–24	21, 14–28	0.101
Baseline TBIL		12.600, 10–16.1	13.400, 10–16.85	0.297
Baseline MONO		0.360, 0.30–0.45	0.400, 0.32–0.46	0.058
Baseline NLR		1.800, 1.36–2.45	1.655, 1.31–2.28	0.231
Baseline PLR		133.000, 109–170	129.000, 106–171	0.890
	Baseline CA153	8.800, 7–12.6	10.000, 6.93–15.0	0.106
	Baseline CEA	1.600, 1–2.3	1.700, 1–2.67	0.345
ECOG score				0.264
	0	251 (98.4)	205 (99.5)	
	1	4 (1.6)	1 (0.5)	
Hypertension disease				0.778
	No	231 (90.6)	185 (89.8)	
	Yes	24 (9.4)	21 (10.2)	
Diabetes				0.051
	No	232 (91.0)	197 (95.6)	
	Yes	23 (9.0)	9 (4.4)	
Cholecystitis				0.113
	No	245 (96.1)	191 (92.7)	
	Yes	10 (3.9)	15 (7.3)	
History of gallstones				0.003**
	No	250 (98.0)	190 (92.2)	
	Yes	5 (2.0)	16 (7.8)	
Peripheral blood circulation				<0.001**
	Poor	88 (34.5)	34 (16.5)	
	Good	167 (65.5)	171 (83.5)	
Sweat excretion				<0.001**
	Normal	227 (89.0)	151 (73.3)	
	Excessive	28 (11.0)	55 (26.7)	
Allergy or not				0.905
	No	242 (94.9)	196 (95.1)	
	Yes	13 (5.1)	10 (4.9)	
Viral hepatitis				0.750
	No	237 (92.9)	193 (93.7)	
	Yes	18 (7.1)	13 (6.3)	
With or without tumor				0.040*
	No	194 (76.1)	139 (67.5)	
	Yes	61 (23.9)	67 (32.5)	
Ki-67				0.921
	Low	88 (34.5)	72 (34. 9)	
	High	167 (65.5)	134 (65.1)	
ER				0.406
	Negative	81 (31.8)	73 (35.4)	
	Positive	174 (68.2)	133 (64.6)	
PR				0.061
	Negative	98 (38.4)	97 (47.1)	
	Positive	157 (61.6)	109 (52.9)	
HER2				0.001**
	Negative	208 (81.4)	141 (68.4)	
	Positive	47 (18.4)	65 (31.6)	

* *p* < 0.05 ** *p* < 0.01

*Abbreviations*: *ALT* alanine aminotransferase, *AST* aspartate aminotransferase, *BMI* body mass index, *ECOG* eastern cooperative oncology group, *ER* estrogen receptor, *GGT* γ-glutamyl transpeptidase, *HER2* human epidermalgrowth factor receptor-2, *Ki-67* antigen identified by monoclonal antibody Ki-67, *MSHFS* moderate to severe hand-foot syndrome, *MONO* monocytes, *NLR* neutrophil-lymphocyte ratio, *NMHFS* none and mild hand-foot syndrome, *PLR* platelet-lymphocyte ratio, *PR* progesterone receptor, *TBIL* total bilirubin

### Results of multivariate logistic regression analysis

The factor of *P* < 0.05 selected by PLD concurrent MSHFS risk single factor analysis was used for unconditional binary multi-factor progressive logistic regression analysis. The results showed that BMI, PLD dose intensity, peripheral blood circulation, sweat excretion, gallstone history, and HER2 status were independent risk factors for the occurrence of MSHFS, with statistical significance (*P* < 0.05). The order of risk degree from high to low was a history of gallstones (OR = 3.403, *P* = 0.030 < 0. 05), sweat excretion (OR = 2.925, *P* < 0.001), peripheral blood circulation (OR = 1.975, *P* = 0.011 < 0. 05), HER-2 (OR = 1.264, P < 0.001), BMI (OR = 1.116, *P* = 0.002 < 0. 01), and BMI (OR = 1.116, P = 0.002). It was seen that high BMI, high-dose PLD, good peripheral blood circulation, substantial sweating, a history of gallstones and HER positivity could promote the occurrence of MSHFS (Table 4).

**Table 4:** Logistic multivariate regression analysis results

Influencing factors	B	OR	95%CI	*P*
BMI	0.110	1.116	1.043–1.194	0.002**
Dose intensity	0.234	1.264	1.190–1.343	<0.001**
Baseline ALT	0.018	1.018	0.992–1.045	0.169
Baseline AST	-0.025	0.975	0.931–1.022	0.290
History of gallstones	1.225	3.403	1.123–10.316	0.030*
Peripheral blood circulation	0.681	1.975	1.169–3.338	0.011*
Sweat excretion	1.073	2.925	1.652–5.178	<0.001**
With or without tumour?	0.364	1.440	0.901–2.299	0.127
HER2	0.680	1.973	1.201–3.242	0.007**
Constant	−11.138	<0.001		<0.001

* *p* < 0.05 ** *p* < 0.01

Enter quantitative data directly, and the classification data is based on the negative group

*Abbreviations*: *ALT* alanine aminotransferase, *AST* aspartate aminotransferase, *HER2* human epidermalgrowth factor receptor-2

### Establishment of logistic regression prediction probability model

Factors with P < 0.05 screened by single factor analysis were used for unconditional binary multivariate logistic regression analysis to construct the PLD concurrent MSHFS risk regression equation. Logit(P) = ln(*p*/1-*p*) = − 11.138 + 0.110*BMI + 0.234*PLD dose intensity + 0.018 * baseline ALT− 0.025* baseline 11 AST 1.225 * gallstone history + 0.681 * peripheral blood circulation + 1.073 * sweat excretion + 0.364 * with or without tumour + 0.680*HER-2 (where p represents the probability that MSHFS occurs, and 1-P represents the probability that MSHFS does not occur). A probabilistic model for predicting the occurrence of HFS in patients with PLD: *P* = 1/1 + exp. (11.138–0.110*BMI0.234*PLD dose intensity-0.018 * baseline ALT+ 0.025* baseline AST-1.225* gallstone history-0.681 * peripheral blood circulation-1.073 * sweat excretion-0.364 *with or without tumour- 0.680*HER-2).

### Hosmer-Lemeshow fit test

The prediction equation model established in this study has good goodness of fit, χ ^2^ = 12.929, 0.114 > 0.05, which indicates that there is no statistically significant difference between the expected frequency derived from the prediction probability and the observed frequency.

### Evaluation of model prediction

1. Accuracy of model prediction

This regression model predicted the occurrence of MSHFS with an accuracy of 72.5%, a sensitivity of 68.4% and a specificity of 75.7%.

**Fig. 2:**
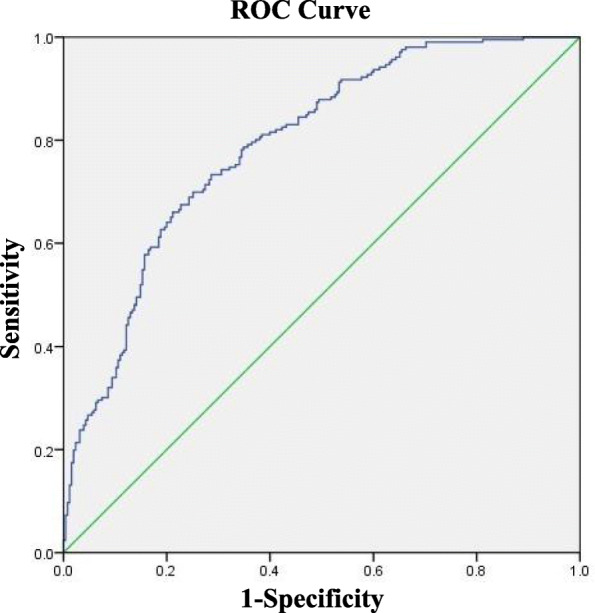
ROC curve

(2) Draw the ROC curve: AUC = 0.791, *P* < 0.01, 95% CI =0.750–0.831 (Fig. 2).

## Discussion

Previous reports have indicated that 45% of ovarian and breast cancer patients develop HFS after PLD chemotherapy, and approximately 4–7% of these patients stop using PLD because of HFS, which affects treatment [9]. Available evidence supports a higher incidence of HFS in Asian people [10]. In a prospective study, a questionnaire survey on the quality of life of 91 patients with HFS was conducted. Among all HFS patients, 47% considered this to be the most painful side effect, and 78% of grade 3 patients had the same opinion [11]. We should pay considerable attention to HFS, especially MSHFS, to find the risk factors for MSHFS as soon as possible, and we should provide corresponding preventive measures to avoid the occurrence of MSHFS.

This study shows that when PLD is used every three weeks, the dose intensity is an independent risk factor that affects the occurrence of MSHFS, that is, the higher the dose intensity is, the greater the likelihood of MSHFS. Several studies have confirmed that the occurrence of HFS is related to the dose intensity and periodic frequency of PLD. Brien et al. validated that during the use of PLD, the emergence of HFS is dose-dependent and periodically frequency-dependent. When the dose intensity of PLD is 40, 45, or 50 mg/m2, the longer the interval between medications is, the better the tolerability. Similarly, if the cycle frequency of PLD is once a week and the dose intensity is 10 mg/m2, it will be better tolerated than the higher concentration of the drug [12].

In this study, the results of unconditional binary multivariate logistic regression analysis showed that peripheral blood circulation and sweat excretion were independent risk factors for the occurrence of MSHFS. The more abundant the peripheral blood circulation and excessive sweat excretion were, the greater the probability of MSHFS. This may be due to the many unique characteristics of the skin of the palms and soles of the feet, including the rapid proliferation of skin cells and the presence of a temperature gradient, a high-density capillary network, exocrine sweat glands, and palms and soles of the feet, which are more likely to be exposed to local factors such as friction and trauma [13–15]. Some people speculate that the palms and soles of the feet are prone to repeated friction or trauma, and there may be richer capillary networks and more blood flow in these areas, while PLD is preferentially located in areas with high vascular permeability, so there may be higher concentrations of PLD in the palms and soles of the feet [16]. There are a large number of secretory glands in the hands and feet, and PLD is excluded by the secretory glands, resulting in HFS in the hands and feet [17]. This may also be one of the reasons why patients with hyperhidrosis are prone to HFS grade 3 reactions [18]. The reason why PLD is easily excreted through sweat glands may be that PLD is encapsulated in pegylated liposomes so that PLD can escape the rapid recognition of macrophages and monocytes. The hydrophilic coating of PLD can accelerate the sweat transport of the PLD reagent and accumulate on the surface of the skin [9]. Compared with common anthracyclines, PLD circulates in the blood longer [19]. There are related theories that after PLD accumulates on the surface of the skin, it can penetrate into the stratum corneum and deeper skin [18]. Therefore, HFS may be more likely to occur in areas with thicker cuticles, such as the palms and soles of the feet [20]. Therefore, before using PLD, patients should be carefully asked about the condition of peripheral blood circulation (temperature of the hands and feet) and the degree of perspiration, which may be beneficial for the prevention and treatment of HFS.

According to related literature reports, PLD is mainly metabolized by the liver and excreted by bile [21]. If patients have poor liver function or poor bile excretion, it can cause the accumulation of PLD in the body, which makes the patient prone to HFS. The study found that baseline ALT, baseline AST and gallstone history were risk factors for MSHFS, and gallstone history was an independent risk factor, which has a significant positive impact on the occurrence of MSHFS. Therefore, before the use of PLD, carefully asking patients whether there is a gallstone-related history, referencing the baseline ALT and AST-related biochemical indicators, may be helpful for the prevention of MSHFS and the relevant adjustments of PLD dose.

At present, there is no final conclusion about the relationship between BMI and HFS. It has been reported that there is no relationship between BMI and HFS risk [22, 23]. However, in a retrospective study of HFS caused by the use of PLD in recurrent ovarian cancer, the researchers found that with the increase in BMI, the number of patients with HFS decreased, but the change was not statistically significant (*P* = 0.19) [24]. There are also clinical studies showing that plasma exposure in patients with higher BMI seems to be lower [25–27]. However, in this retrospective study, BMI was an independent risk factor for MSHFS, and the higher the BMI was, the higher the incidence of MSHFS. The reasons for this difference may be related to the following factors: different cancers, different statistical and stratified types, and different sample sizes. Therefore, in future research, it is necessary to further develop the relationship between BMI and MSHFS to provide a basis for the prevention and treatment of MSHFS.

The proportion of HER2-positive patients in the MSHFS group was higher than that in the NMHFS group, with statistically significant differences (*P* value < 0.01). The results of unconditional binary multivariate logistic regression analysis showed that HER2 positivity was an independent risk factor for MSHFS, that is, HER2-positive patients were more likely to develop MSHFS than HER2-negative patients. However, there are no other studies showing that HER2 is related to the induction of MSHFS. Therefore, the results in this study need to be confirmed by further studies.

**Fig. 3:**
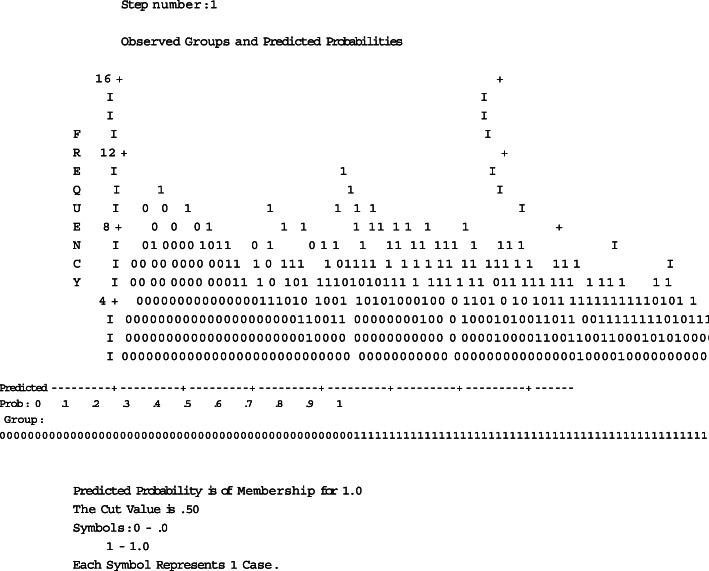
Observed group and predicted probability graph

Through binary logistic regression analysis, the prediction equation was established, and the formula for predicting the probability of MSHFS occurrence was obtained. In typical clinical work, breast cancer patients treated with neoadjuvant and adjuvant chemotherapy can be assessed for whether there are corresponding risk factors, the *P* value formula and P value can be obtained, and then the possible probability of MSHFS occurrence might be speculated. In the Fig. 3, it can be seen that: the Cut Value is 0.5, where 0.5 is the cut value, and the predicted probability is greater than 0.5, indicating that the probability of the patient “has MSHFS” is relatively large, and less than 0.5 indicates the patient” The probability of occurrence of MSHFS is relatively small. If the P value is greater than 0.5, it should be highly suspected that MSHFS will occur, and corresponding preventive measures should be given. Such as: Patient education and prevention; Physical cooling such as local cooling, Antiperspirant for external use, the use of steroid hormones, urea ointment and other drugs for prevention. Appropriate reduction of the dose, studies have shown that regardless of the dosing interval, when the PLD dose intensity is maintained at 10 mg/m2 per week, the effect is better and the adverse reactions are tolerable; when the PLD dose does not exceed 10 mg/m2 per week, most HFS Mild to moderate levels, and to a certain extent can avoid the occurrence of potential HFS. This approach may provide advanced prediction and prevention, may reduce the occurrence of MSHFS, and may improve the quality of life and treatment effect of patients.

The shortcoming of this article is that this study is a single-centre retrospective analysis. The results of the research may be biased and need to be confirmed by multi-centre, prospective research. Nevertheless, to our knowledge, this study is one of the largest studies on MSHFS to date that identified risk factors for breast cancer patients who develop MSHFS, not just reporting the incidence of MSHFS. We have also established a prediction model of MSHFS occurrence that may provide the possibility of preventing MSHFS in advance. However, this model has not been validated by the clinical validation cohort, and clinicians need to use it to further confirm its applicability.

## Data Availability

The datasets used and/or analyzed during the current study are available by contacting wenhui zhao by email (zhaoteam1@163.com) on a reasonable request.

## Abbreviations

ALT: Alanine aminotransferase
AST: Aspartate aminotransferase
BMI: Body mass index
ECOG: Eastern cooperative oncology group
ER: Estrogen receptor
GGT: γ-glutamyl transpeptidase
HER2: Human epidermalgrowth factor receptor-2
Ki-67: Antigen identified by monoclonal antibody Ki-67
MSHFS: Moderate to severe hand-foot syndrome
MONO: Monocytes
NLR: Neutrophil-lymphocyte ratio
NMHFS: None and mild hand-foot syndrome
PLR: Platelet-lymphocyte ratio
PR: Progesterone receptor
TBIL: Total bilirubin
